# Occult metastases and survival of lung cancer by clinical diagnosis and CT screening: A simulation study

**DOI:** 10.1371/journal.pone.0313544

**Published:** 2025-01-03

**Authors:** Xing Chen, Ghulam Muhammad Kanhar, Songli Hu, Chaomin Wu, Guanqun Chao, Mengqi Jing, Fengjiang Zhang, Millennia Young, Marek Kimmel, Liying Chen, Olga Y. Gorlova

**Affiliations:** 1 Department of Medicine, Zhejiang Sir Run Run Shaw Hospital, Zhejiang University, Hangzhou, China; 2 Department of Biomedical Engineering, Key Laboratory of Biomedical Engineering of Ministry of Education of China, Zhejiang University, Hangzhou, Zhejiang, China; 3 Department of Anesthesiology, Second Affiliated Hospital, Zhejiang University, Hangzhou, Zhejiang, China; 4 Department of Anesthesiology, The Fourth Affiliated Hospital, International Institute of Medicine, Zhejiang University School of Medicine, Yiwu, China; 5 Department of Medicine Epidemiology and Population Sciences, Baylor College of Medicine, Houston, Texas, United States of America; 6 Human Health and Performance Directorate / Biomedical Research & Environmental Sciences Division, NASA Johnson Space Center, Houston, Texas, United States of America; 7 Departments of Statistics and Bioengineering, Rice University, Houston, Texas, United States of America; European Institute of Oncology: Istituto Europeo di Oncologia, ITALY

## Abstract

**Objectives:**

It is significant to know how much early detection and screening could reduce the proportion of occult metastases and benefit NSCLC patients.

**Methods:**

We used previously designed and validated mathematical models to obtain the characteristics of LC in the population including undetectable metastases at the time of diagnosis. The survival was simulated using the survival functions from Surveillance, Epidemiology and End Results (SEER) data stratified by stage.

**Results:**

Based on the simulations, 35.3% of patients diagnosed with stage N0M0 and 56.9% of those diagnosed with stage N1M0 had nodal or distant metastases that were not discovered at the time of diagnosis. Among clinically detected Stage I lung cancers with tumor diameter 1–2 cm, 78% were true stage N0M0 (no occult metastases) while it was only 37% for patients with tumor diameters of 2–3 cm. This size threshold can be translated into a 0.75-year the “window of opportunity” for the curable disease. In a comparative analysis of two simulated groups of individuals: (1) clinically diagnosed (2) diagnosed by screening with a varying screening frequency (quarterly, biannual, annual and biennial), it was estimated that, once the screening intervals become shorter, substantially more cancers are found, but at an expense of a higher radiation exposure. The simulation projected that the mortality reduction in screened patients depending on the frequency, ranged from 15.04% to 18.82%.

**Conclusions:**

The probability of occult metastases significantly increases when the primary tumor exceeds 2 cm in diameter. Effective screening measures that detect smaller tumors will considerably benefit asymptomatic LC patients.

## 1. Introduction

Lung cancer remains a disease with a high mortality, even when diagnosed at an early stage, particularly since asymptomatic metastases are often present at the time of diagnosis [1–4]. Although screening helps find early-stage disease, the debate on the benefits of LC screening has been continuing for decades. Occult tumors are tumors the presence and location of which cannot be identified during standard clinical evaluation [5–8]. The presence of occult metastases in lymph nodes, pleura, or distant sites which are hard to find by standard diagnosis procedures in clinical practice are a likely reason for poor outcomes in early stage non-small cell lung cancer (NSCLC) [9]. The American College of Surgeons Oncology Group (ACOSOG) Z0040 trial revealed that 22% of patients with N0 disease had occult nodal metastases associated with decreased overall and disease-free survival [10, 11]. It is increasingly important to understand how well early LC detection by screening can reduce the proportion of patients with occult metastases. We compared the probability of occult metastases in clinically detected and screened populations using a method to predict the true stages by modeling the natural history and detection of NSCLC.

## 2. Materials and methods

In this article, we use a microsimulation-based LC natural history and detection model framework [12], which was previously calibrated to Surveillance, Epidemiology and End Results (SEER), to estimate the presence of occult metastases and survival in a population screened for LC. We also apply the procedure to gain understanding of the potential of screening to detect early non-metastatic lung cancers, as a function of primary tumor size at detection. Individuals aged 55–77 with a smoking history of 30 or more pack-years were considered eligible for screening, and we applied varying screening intervals in the model framework. We also applied an updated high-risk group definition as per most recent NCCN guidelines [13], including individuals aged 50 or older with 20 or more pack-years of smoking.

### 2.1. Model framework

**Fig 1:**
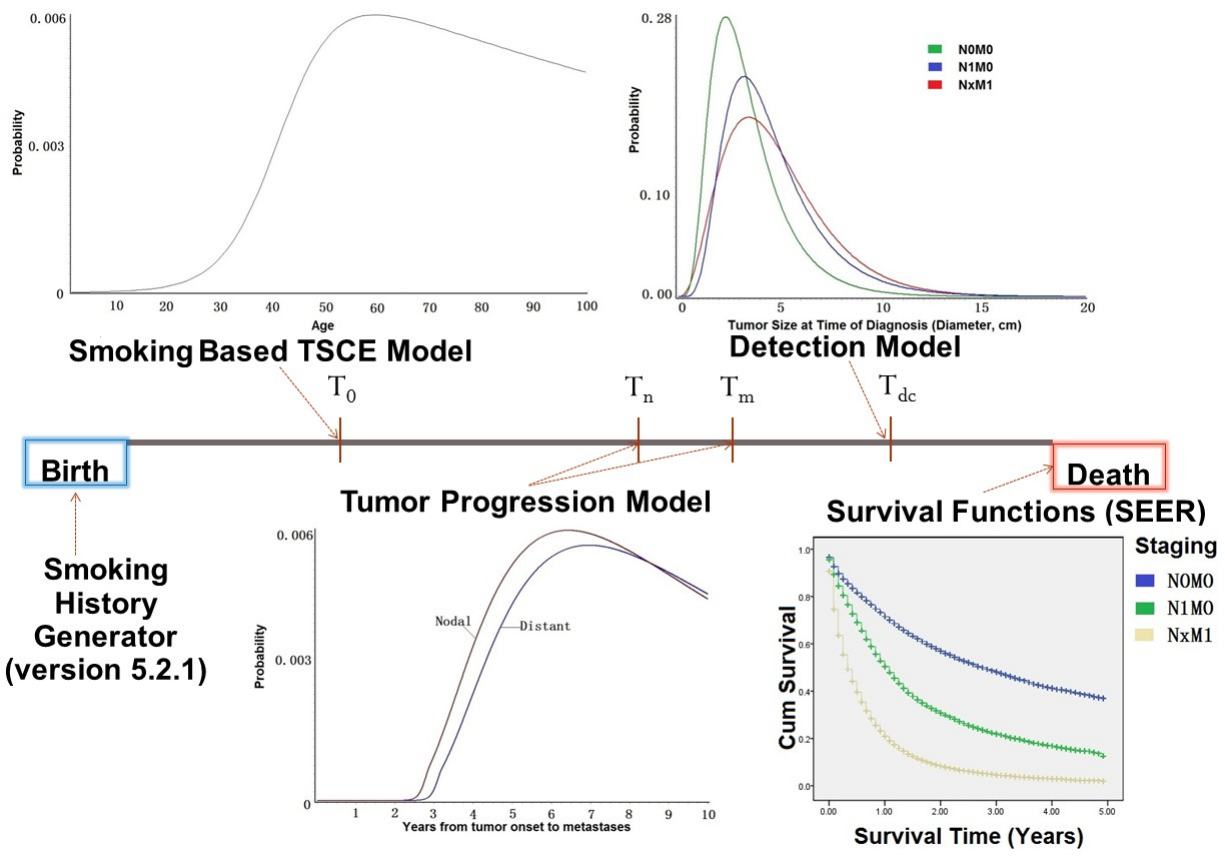
The diagram of micro-simulation using the smoking based two-stage clonal expansion model framework.

In this framework, we jointly apply the two-stage clonal expansion (TSCE) carcinogenesis model, tumor-progression and detection models, and stage- and primary tumor size-specific survival functions derived from LC patients diagnosed from 2004 to 2008 using data from SEER as depicted in Fig 1. We construct the individuals timeline from his/her birth to the time of lung cancer initiation (*T*_*0*_, age at tumor onset), progression (*T*_*n*_ and *T*_*m*_, ages at nodal and distant metastasis), detection (*T*_*dc*_, age at detection), and death by combining models of carcinogenesis, tumor progression (growth and metastasis), detection, and survival.

The smoking based TSCE carcinogenesis model [14] combined with the data on the patients’ smoking duration and intensity generated by the smoking history generator (SHG, version 5.2.1) [15–17] is used to predict the occurrence of lung cancer within a person’s lifetime, as well as to calculate the age of the patient at lung cancer initiation. The SHG is a tool developed by the National Cancer Institute based on historical smoking patterns. It simulates individual life/smoking histories such as the age at smoking initiation, cessation, and smoking intensity (cigarettes per day), by gender and birth cohort, initially comprising the period from 1890 to 1984 for the year of birth [17] and subsequently updated to include more contemporary cohorts [18]. The generated smoking histories served as input for the TSCE model that calculated the age at LC initiation. We used the number of live births in the US from the Centers for Disease Control and Prevention to determine the exact number of people that needed to be simulated in each year. The tumor growth and detection models are applied jointly to predict the age, tumor size, and disease stage at the time of diagnosis for the modeled individual. We assume that a tumor grows exponentially with a growth rate λ, which obeys Gamma distribution with parameters of shape *K* and scale *θ*.$F_{n}\left(s\right)=1-e^{-\frac{\mu_{n}}{\xi +1}*S^{\left(\xi +1\right)}}$, and $F_{m}\left(s\right)=1-e^{-\frac{\mu_{m}}{\xi +1}*S^{\left(\xi +1\right)}}$ were the metastases models, where ${\bar{F}}_{\text{n}}$, ${\bar{F}}_{\text{m}}$ are the survival functions for metastasis (nodal or distant), *ξ* is the detachment rate of primary tumor, *μ*_*n*_ and *μ*_*m*_ are the transfer and deposition rates of cancer cells to nodal and distant sites, and *S* is the primary tumor volume. We also assume that the hazard of tumor detection depends linearly on the volume of the tumor, and denote the efficiency of detection by tumor volume as η and stage-dependent offset parameters as *W*_*0*_, *W*_*1*_ and *W*_*2*_. The resulting c.d.f. *D*_*p*_ (*s*_*p*_), *D*_*n*_ (*s*), and *D*_*m*_ (*s*) of detection by volume of the primary tumor and nodal and distant metastases and the estimates of the model parameters are given in our previous work [12]. S1 Table in S1 File shows the parameters of tumor progression and detection models that were used in the simulation framework.

### 2.2. Occult metastases

Assuming that tumors smaller than 0.618 mm in diameter, corresponding to the spherical volume of 0.000124 cm^3^, cannot be detected by CT, we set the detectable size for primary tumor, nodal metastases, and distant metastases at 0.000124 cm^3^. We chose this number based on the fact that the smallest reconstruction slice interval for CT is about 0.3 mm. After assuming that data at 3 points or slices (with 2 slice interval) could be used to discover the abnormal lung tissue, we arrived at the above-mentioned approximation of the limit of tumor detection [19, 20]. Thus, nodal and distant metastases larger than a single malignant cell (volume of 1×10^−9^ cm^3^) and smaller than 0.000124 cm^3^ are considered occult metastases.

### 2.3. Modeling of survival

The survival was simulated using the survival functions derived from SEER data (2004–2008) stratified by tumor size and stage (IA, IB, IIA, IIB, IIIA, IIIB and IV). The years of survival after the time of diagnosis were determined using the inverse stage and size-specific survival function Lung cancer staging is based on the detected primary tumor size (PTS), nodal metastases presence and size (NMS), and distant metastases presence (DMP) (S2 Table in S1 File), and each simulated person was staged accordingly. For Stage I tumors, Survival was further stratified by the size of primary tumor using the estimates given in [21].

### 2.4. Screening unperturbed lung cancer population

We also generated a lung cancer population assuming the symptomatic detection does not take place and all detection is due to periodic screening. Basic information of the generated population included age at tumor onset, age at smoking initiation, age at smoking cessation, smoking intensity, age at nodal and distant metastasis, tumor growth rate, and size of primary tumor, nodal and distant metastasis at each simulated year. We applied quarterly, biannual, annual and biennial screening to this population to generate a simulated screen-detected population. The analysis to estimate mortality reduction was performed as a comparison between unperturbed data (baseline) and the simulated data. The screening specific recorded timeframe was from 1979 to 1999. Following the previous published definitions [22, 23], the difference in the number of LC deaths between no–screening (baseline) and screening scenarios (the number of LC death avoided by screening) divided by the deaths in baseline gives the estimates of mortality reduction that could have been achieved by lung cancer screening from 1979 to 1999.

## 3. Results

Nodal and distant metastases smaller than 0.000124 cm^3^ are considered undetectable in the simulation. Their records were stored to obtain the distributions of occult nodal and distant metastases. 64.7% of patients diagnosed with the stage N0M0 had no hidden metastasis, while 9.3% and 26.0% of these patients respectively had nodal and distant metastases that were not discovered at the time of diagnosis (Table 1). Among N0M0 patients with a primary tumor no larger than 1 cm in diameter, 17.8% and 11.7% had unobservable nodal and distant metastasis at the time of diagnosis, respectively. There was a considerable proportion (38.7%) of undiscovered distant metastasis in patients with a primary tumor no larger than 3 cm, while the proportion dropped dramatically to 7.0% in patients with a primary tumor larger than 3 cm. The fact that the tumors diagnosed at a larger size tended to have a lower metastatic burden implies that those tumors have lower metastatic potential and therefore are not detected until the primary tumor reaches the larger size. 56.9% of patients at stage N1M0 had hidden distant metastasis. Among patients with hidden distant metastases, a high proportion (over 80%) had a primary tumor smaller than 3 cm (Table 1).

**Table 1:** Distributions of occult nodal and distant metastases in the simulated patients symptomatically detected with lung cancer (1988–1999) with stage *N0M0*, *N1M0* and *M1* stratified by tumor size.

Observed stage	True stage	Total	Groups of predicted lung cancer tumor size					
			TS*≤0.5	0.5<TS≤ 1	1<TS≤ 1.5	1.5<TS≤ 2	2<TS≤ 3	TS >3
N0M0		(N = 380017)	(N = 3040)	(N = 18301)	(N = 47534)	(N = 59685)	(N = 100031)	(N = 151426)
N0M0, n (%)		245871 (64.7)	2860 (94.1)	12151 (66.4)	20749 (43.7)	21735 (36.4)	53346 (53.3)	135030 (89.1)
N1M0, n (%)		35342 (9.3)	122 (4.0)	3704 (20.2)	12000 (25.2)	9583 (16.1)	4084 (4.1)	5849 (3.9)
M1, n (%)		98804 (26.0)	58 (1.9)	2446 (13.4)	14785 (31.1)	28367 (47.5)	42601 (42.6)	10547 (7.0)
N1M0**		(N = 321221)	(N = 813)	(N = 5500)	(N = 16827)	(N = 24513)	(N = 62866)	(N = 210702)
N1M0, n (%)		138412 (43.1)	150 (18.5)	506 (9.2)	3512 (20.9)	4769 (19.2)	12084 (19.2)	117391 (55.7)
N1M1, n (%)		182809 (56.9)	663 (81.5)	4994 (90.8)	13315 (79.1)	19744 (80.8)	50782 (80.8)	93311 (44.3)
M1		(N = 732786)	(N = 2058)	(N = 13450)	(N = 33883)	(N = 54203)	(N = 138575)	(N = 490617)
M1, n (%)		732786 (100)	2058 (100)	13450 (100)	33883 (100)	54203 (100)	138575 (100)	490617 (100)

*TS, Primary tumor size (cm) in diameter

**In SEER data, 7208 were N0M1, which is 9.7% of 74109 that had *N* and *M* staged. This stage is not modeled.

The simulated no-intervention scenario depicted in Table 2 provided an important observation of the existence of a crucial point in the primary tumor size, which, when exceeded, results in a high likelihood of a metastatic stage. While among the tumors with sizes 1–2 cm, 78% are stage N0M0, among the tumors with sizes in the 2–3 cm range, only 37% are N0M0. At the same time, the proportion of M1 tumors increases from 14% to 41%.

**Table 2:** Distributions of true nodal and distant metastases in simulated lung cancer patients* without any diagnostic intervention at years 1978, 1988, and 1998, stratified by tumor size.

Year	True stage	Total	Groups of predicted lung cancer tumor size					
			TS**≤0.5	0.5<TS≤ 1	1<TS≤ 1.5	1.5<TS≤ 2	2<TS≤ 3	TS >3
1978		(N = 988701)	(N = 739313)	(N = 59103)	(N = 33283)	(N = 22865)	(N = 31397)	(N = 102740)
N0M0, n (%)		857535 (86.7)	739008 (100.0)	57214 (96.8)	28642 (86.0)	15112 (66.1)	11489 (36.6)	6070 (5.9)
N1M0, n (%)		27127 (2.7)	125 (0.0)	755 (1.3)	1824 (5.5)	3007 (13.1)	6895 (22.0)	14521 (14.1)
M1, n (%)		104039 (10.5)	180 (0.0)	1134 (1.9)	2817 (8.5)	4746 (20.8)	13013 (41.4)	82149 (80.0)
1988		(N = 1143627)	(N = 849625)	(N = 69322)	(N = 39152)	(N = 27345)	(N = 37115)	(N = 121068)
N0M0, n (%)		988933 (86.5)	849297 (100.0)	67085 (96.7)	33684 (86.0)	18202 (66.6)	13571 (36.6)	7094 (5.9)
N1M0, n (%)		31285 (2.7)	141 (0.0)	875 (1.3)	2143 (5.5)	3594 (13.1)	7999 (21.6)	16533 (13.7)
M1, n (%)		123409 (10.8)	187 (0.0)	1362 (2.0)	3325 (8.5)	5549 (20.3)	15545 (41.9)	97441 (80.5)
1998		(N = 1209745)	(N = 894018)	(N = 74101)	(N = 41871)	(N = 29208)	(N = 39941)	(N = 130606)
N0M0, n (%)		1042485 (86.2)	893662 (100.0)	71731 (96.8)	36183 (86.4)	19310 (66.1)	14172 (35.5)	7427 (5.7)
N1M0, n (%)		33378 (2.8)	122 (0.0)	962 (1.3)	2186 (5.2)	3833 (13.1)	8661 (21.7)	17614 (13.5)
M1, n (%)		133882 (11.1)	234 (0.0)	1408 (1.9)	3502 (8.4)	6065 (20.8)	17108 (42.8)	105565 (80.8)

*Individuals in whom lung cancer is developing but is not diagnosed and who are alive or have died of other cause; the assumption is that symptoms never develop regardless of the stage

**TS, Primary tumor size (cm) in diameter

**Fig 2:**
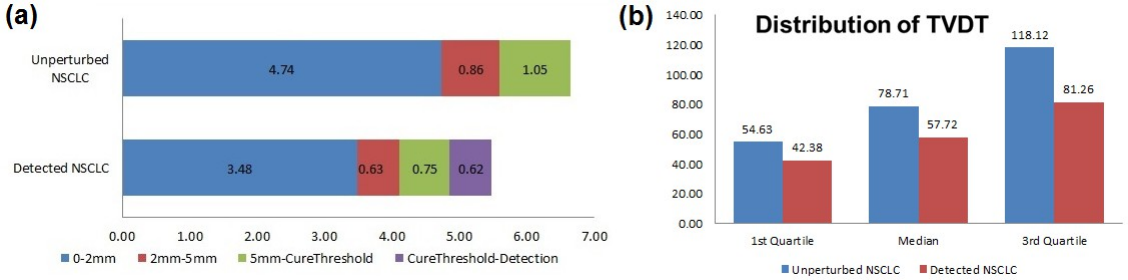
(a) NSCLC progression timeline (in years from the first malignant cell) where the median lengths of the following time intervals are presented: Progression from 0 to 2 mm, 2 to 5 mm, 5 mm to cure threshold, and cure threshold to detection. Two versions of the timeline refer to the unperturbed disease (top) and detected disease (bottom), respectively. (b) Median and the first and third quartile of the tumor volume doubling time (TVDT) in the unperturbed versus detected NSCLC.

Screening for asymptomatic tumors was simulated in the framework of our model when not applying the symptomatic detection module, which results in the characteristics of all tumors (whether they would have produced symptoms or not) being recorded. This does not occur in reality, but becomes relevant if screening is performed. Table 2 presents the distributions of nodal and distant metastases in simulated lung cancer patients, who developed disease but were assumed not to develop any symptoms in their lifetime. In this unperturbed natural history simulation, there was no secular trend observed. 86% of patients had a stage N0M0 disease, of which about 86% had a primary tumor smaller than 0.5 cm in diameter and 80% of the tumors would have been undetectable by imaging because of screening detection limits. Most of the patients with nodal and distant metastases (N1M0 or M1) had a detectable primary tumor (tumor size ≥ 1cm in diameter). Moreover, tumor size by stage distribution did not show variation by age. In Fig 2, we present the timeline estimated using our model, whereby two scenarios are considered. The first one (“unperturbed”) implies no intervention in the course of the disease. The second concerns disease detected by usual clinical practice (through symptoms). The “window of opportunity” for the curable disease has a 0.75-year width, if it is assumed that the smallest detectable tumors have 5 mm size.

Detection at an early stage is vital to reducing the unacceptably high rate of lung cancer fatality. However, to estimate the right frequency of screening it is important to take into account cost and exposure to radiation. In this study we applied varying screening frequency (quarterly, biannual, annual and biennial) to high-risk individuals as defined by NCCN recommendations. We simulated two groups of high-risk individuals. Group 1 consist of high risk individuals aged 55 to 77 years having smoking history of 30 or more pack-years who are either current smokers or quit smoking in the past 15 years. Group 2 consists of high-risk individuals aged 50 to 74 years with smoking history of 20 or more pack-years, either current smokers or those who quit smoking in the past 15 years. Furthermore, following the recent NCCN recommendation [13], we increased the smoking cessation duration from 10 to 15 years. It appeared that the screening interval for a given duration of cessation and pack-year scenario had no significant effect on age at diagnosis, number of CT screens and overall survival years of lung cancer patients of Group 1 and Group 2 as shown in Table 3. While considering the screening interval in each group there is a significant difference in age at diagnosis, number of CT scans and survival years of lung cancer patients. In comparison to baseline, the model-simulated screening leads to an earlier age at diagnosis, longer survival, and mortality reduction.

**Table 3:** Comparative analysis of SEER overall, baseline, and two simulated groups that differ by smoking history and are screened at different intervals; Stage 1 only.

Smoking History		Screening start & end age	Quit year	Screening Frequency*	Age at diagnosis		Survival year		Age at Death		CT Screen LC**
					Mean ± SD	Median ± IQR	Mean ± SD	Median ± IQR	Mean ± SD	Median ± IQR	
SEER					68.23±9.77	69(62~75)	5.33±4.66	4.16(1.41~8.00)	73.56±9.50	74.50(67.66~80.33)	
Baseline					66.02±14.19	67(57~76)	5.68±6.26	3.29(1.24~7.86)	71.70±14.60	73.00(63.01~81.77)	0
Group 1	30	55–77	10	0.5	66.03±10.01	66(59~73)	13.33±10.87	9.51(4.38~22.00)	79.36±12.76	79.00(71.00~88.00)	88
	30	55–77	10	1	65.47±9.83	66(59~72)	13.63±10.95	10.00(4.77~22.08)	79.10±12.75	78.77(70.75~87.78)	175
	30	55–77	10	2	65.16±9.78	65(59~71)	13.72±10.91	10.00(4.85~22.18)	78.88±12.77	78.50(70.31~87.50)	330
	30	55–77	10	4	64.95±9.73	65(59~71)	13.75±10.93	10.00(4.80~22.25)	78.73±12.72	78.25(70.25~87.25)	643
	30	55–77	15	0.5	65.83±10.00	66(60~72)	13.36±10.95	9.47(4.31~22.00)	79.20±12.82	79.00(70.82~88.00)	100
	30	55–77	15	1	65.85±9.84	66(59~72)	13.61±10.91	10.00(4.86~22.03)	79.31±12.75	79.00(71.00~88.00)	186
	30	55–77	15	2	65.33±9.75	65(59~72)	13.75±10.95	10.00(4.86~22.33)	79.08±12.71	78.54(70.51~87.50)	352
	30	55–77	15	4	65.19±9.74	65(59~72)	13.79±10.92	10.00(4.87~22.49)	78.98±12.79	78.50(70.39~87.50)	687
Group 2	20	50–74	10	0.5	64.59±10.38	65(58~71)	13.91±11.17	10.00(4.68~23.00)	78.50±13.03	78.00(70.00~87.00)	134
	20	50–74	10	1	64.51±10.28	65(57~72)	14.12±11.12	10.01(5.00~23.00)	78.63±13.02	78.04(70.00~87.00)	240
	20	50–74	10	2	64.21±10.25	64(57~71)	14.20±11.12	10.50(5.00~23.10)	78.41±13.02	78.03(69.86~87.00)	457
	20	50–74	10	4	63.75±10.17	64(57~72)	14.33±11.18	10.60(5.00~23.29)	78.08±13.01	77.78(69.38~86.75)	930
	20	50–74	15	0.5	64.79±10.37	65(58~72)	13.85±6.26	10.00(4.67~23.00)	78.65±13.02	78.27(70.00~87.14)	141
	20	50–74	15	1	64.60±10.22	65(58~71)	14.06±11.13	10.01(5.00~23.00)	78.66±12.95	78.24(70.00~87.08)	264
	20	50–74	15	2	64.19±10.27	65(57~71)	14.16±11.12	10.50(5.00~23.00)	78.35±13.06	78.00(69.64~87.00)	496
	20	50–74	15	4	63.98±10.23	64(57~71)	14.30±11.15	10.50(5.00~23.29)	78.29±13.02	78.00(69.61~87.00)	985

*screening interval: 0.5 denotes 1 CT scan in two years (biennial), 1 denotes 1 CT scan in 1 year (annual), 2 denotes 2 CT scans in one year (biannual), and 4 denotes 4 CT scans in one year. The data used in table is the representation of stage 1.

** The number of screen to find lung cancer and the estimated cost of single CT screening is $3074 [24].

**Fig 3:**
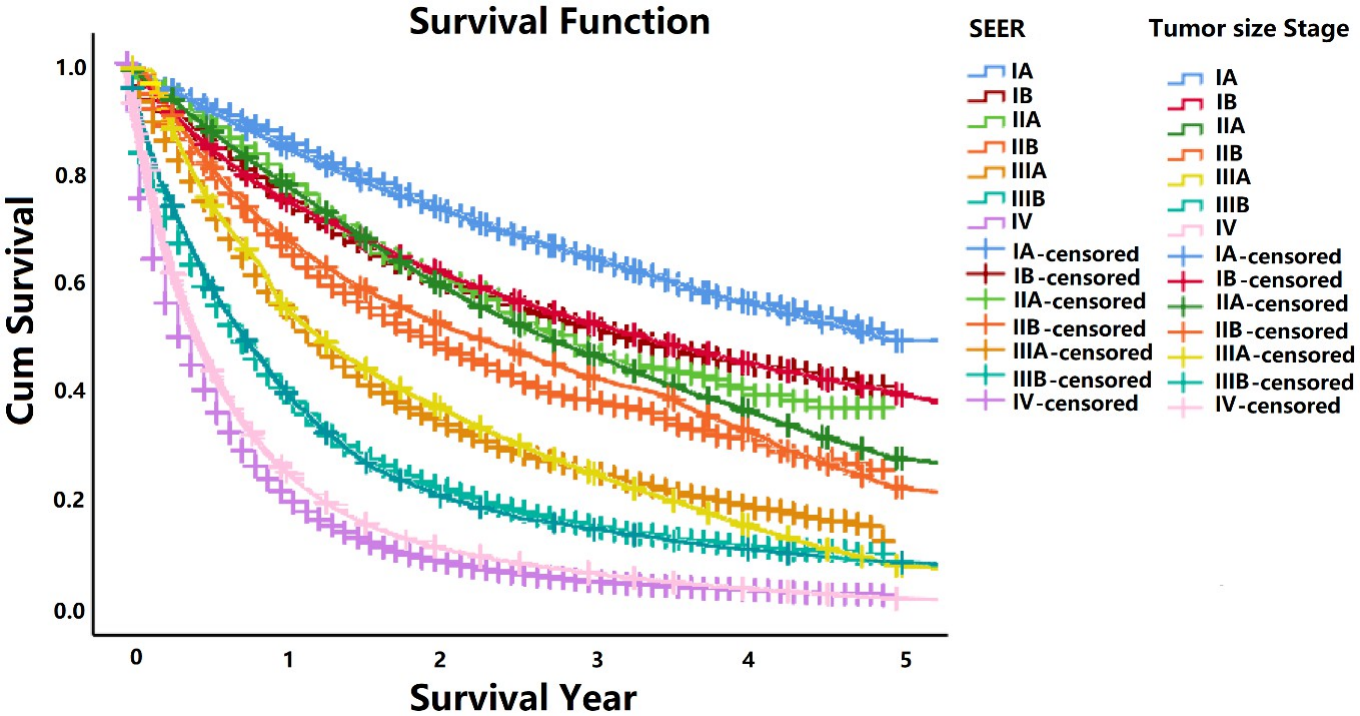
The comparative analysis of five-year survival between SEER and tumor size-based stages simulated screening data.

**Fig 4:**
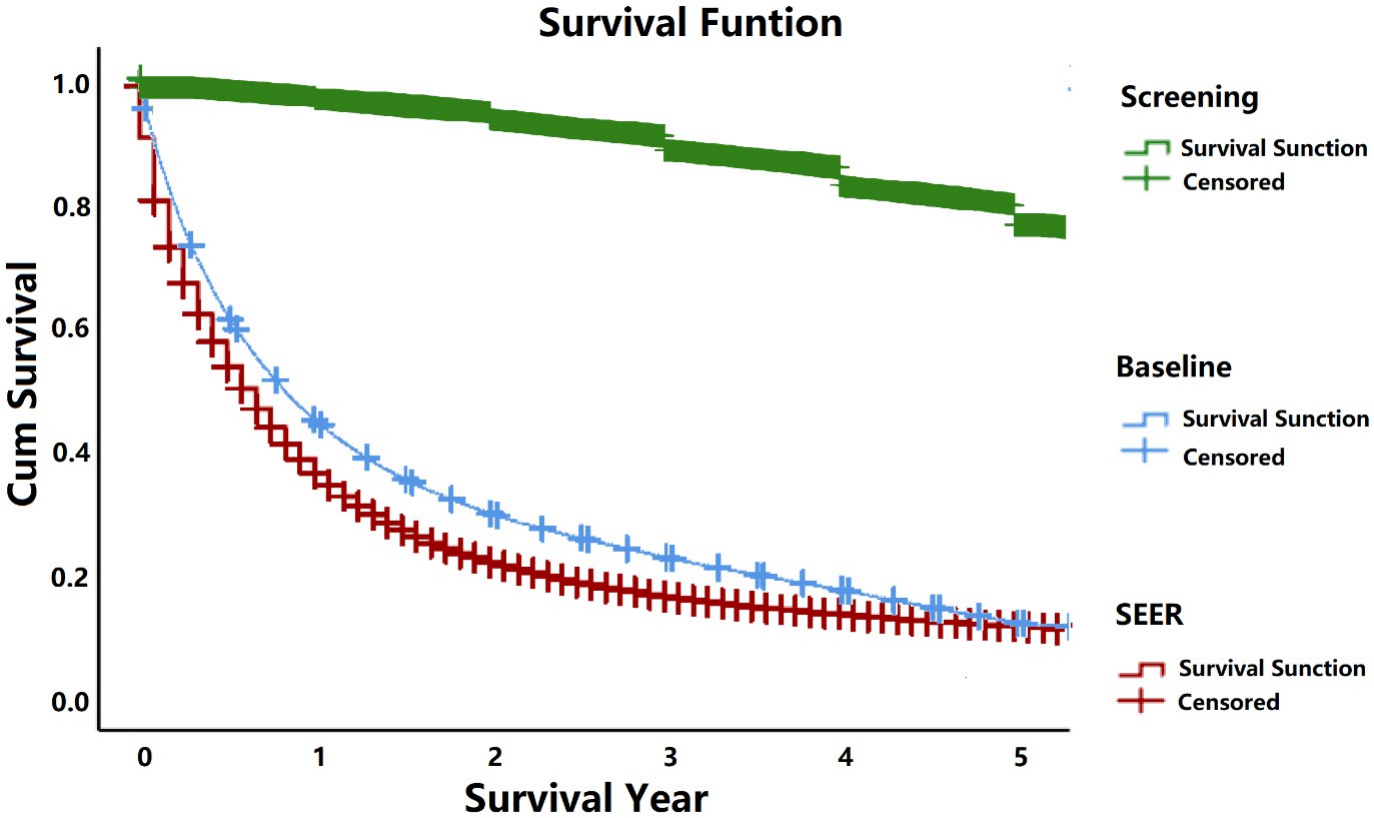
The comparative analysis of an overall five-year survival between SEER, baseline and screening simulated data.

Survival Function: We validated the fitted model by comparing the stage-specific survival in SEER and simulated data, which were comparable as shown in Fig 3. Furthermore, we performed the comparative analysis between SEER and stage simulated data without tumor size information. The staging information without tumor size is shown in S1-S6 Figs in S1 File. However, to further validate the model we used another methodology from published paper not based on SEER to estimate the survival function specific to stage 1 only [21]. We used the calculated estimates in the simulation model and extracted the data for the analysis. When comparing an overall distribution of survival of the SEER, baseline and screening simulated data distribution by selecting screening parameters of group 1 with screening frequency once a year and smoking cessation time fifteen years as shown in Table 3, we observed a major difference in survival plots as shown in Fig 4, owing to a difference in the stage distribution between the screened and unscreened populations.

Survival and mortality reduction: The analysis of simulated data estimated that screening strategies resulted a significant decrease in LC mortality with significant survival years. The simulated data estimated the mortality reduction following screening interval group 1: quarterly 15.18% (95% CI 10.87%±19.50%), biannual 15.49% (95% CI 11.15%±19.84%), annual 15.43% (95% CI 10.77%±20.11%), biennial 15.04% (95% CI 10.73%±19.36%) and group 2: quarterly 18.71% (95% CI 13.44%±23.98%), biannual 18.43% (95% CI 13.24%±23.69%), annual 18.82% (95% CI 13.25%±24.39%), biennial 18.39% (95% CI 13.02%±23.77%). We assessed the mortality reduction from 1980 to 1999. The simulated data screening intervals projected annual mortality reduction for group 1 which peaked in 1980 at 33.58% and decreased to 2.06% by 1999 and group 2 in 1980 at 41.07% and decreased to 3.69% by 1999 are shown in S7-S9 Figs in S1 File. Our study estimates a decrease in mortality and demonstrates the potential of lung cancer screening to reduce LC mortality. Observing difference between group 1 and group 2 screening intervals based on data and description presented in Table 3 we think group 1 screening interval is considerably better option in terms of time, cost, and reducing exposure to radiation.

## 4. Discussion

A validated mathematical model for simulating the progression and detection of LC at the individual level was used to obtain the characteristics of LC in a population at the time of diagnosis. Detection threshold of CT scan was assumed to determine undetectable metastases. The survival was simulated using the survival function from Surveillance, Epidemiology and End Results (SEER) data stratified by stage, and for Stage I tumors, further stratified by size [21].

Screening detection was applied to the unperturbed LC population (without any diagnostic interventions). The tumor size- and stage-specific survival was similar in the screened and unscreened populations as shown in Fig 3, but the tumor size and stage distributions in these two populations were very different. The primary tumor size and stage have been changing rapidly with the passage of time and new research approaches [25]. Keeping in mind the current and proposed stage groups as per TNM subsets [26], our model is accurate, the variation difference between SEER tumor size based stage simulated data especially for the survival is acceptable as seen in Fig 3. In the presence of screening tumors are detected earlier than in its absence. As we can see in Table 3, the screening starts earlier than actual median age at lung cancer onset, T_0_, of the baseline and SEER, and this difference case would remain the same for T_n_, T_m_ and T_dc_ in the model. The survival is consistent with that of screen-detected LC as it was in previously published LC screening study [27]. While screening does not improve the survival time if a case is detected with a metastasized tumor the cases screened at an early curable stage are more likely to go through surgeries and have a vital chance to be treated successfully and get additional years of life. The screening strategies help get additional years of life and considerably reduces LC mortality in comparison to other current strategies [28–30]. To check the effectiveness of our screening model we performed a comparative analysis of five-year and fifteen-year survival difference between SEER, baseline and tumor size based simulated screening data. (The details are in S6 Fig in S1 File). The lung cancer spontaneous detection in presence of nodal or distant metastases is much more likely than when only the primary tumor is present (*W1*, *W2 >> W0*), but finding small metastases is more complicated than the detection of primary tumor only, in clinical practice. Our model predicts mean duration from tumor onset to detection consistent with a previous progression study [31]. Among the tumors detected, the mean size of the nodal or distant metastases was less than 1 cm in diameter, which is significantly smaller than suggested by other projections based on screening data for different cancers [32, 33].

Comparison with other Studies: Recently, Lin and Plevritis [34] published the estimates of the disease progression timeline for non-small cell lung cancer (NSCLC; their Fig 4c), based on a computational model with parameter estimates obtained using simulation-based likelihood maximization. The model hypotheses differ somewhat from ours but it is interesting to compare the predictions and to discuss the differences. The most interesting comparison is that the “window of opportunity” for the curable disease has 0.6-year width in Lin and Plevritis (2012) versus 0.75-year width in our study (Fig 2), if it is assumed that the smallest detectable tumors have 5 mm size. If it is assumed that the smallest detectable size is 2mm, then the window of opportunity becomes 2.1 year in Lin and Plevritis (2012) versus 1.38 in our paper. Numbers do not seem radically different; however, they represent opposite directions if the smallest detectable size is varied. These differences can be traced back to differences in volume doubling times. Lin and Plevritis estimate the median volume doubling time as 134 days, whereas the corresponding number in our paper is 79 days. It seems that this latter difference is due to the fact that Lin and Plevritis assume the starting tumor volume to be 1 mm^3^, whereas ours is that of a single cell. Another likely reason is a difference in assumed metastatic rates. However, there are no estimates presented in Lin and Plevritis (2012).

Screening Intervals: LC detection at an early stage has been an elusive goal for decades. While screening is a really hectic and costly process, setting an optimal screening interval could reduce the cost without compromising the survival of LC patients. To address the benefits and harms of screening we compared two simulated data groups of high risk individuals with different recommended screening intervals [35–40]. Previously, de Koning et al. [41] estimated further harms and benefits of LC screening by using 5 models calibrated to NLST and PLCO and presented best case scenario of annual screening age 55 to 80 years. The previous studies [42–47] proposed annual screening strategies and evaluated the harms and benefit of lung cancer screening based on risk factors with start age and end age at screening from 50 or 55 to 74 or 77 or 80 years.

In this study the comparative analysis of two simulated data groups (Table 3) explains that the group 1, biennial screening starting at age at 55 years and ending at age 77 years with smoking history of least 30 pack per years and quit year minimum 10 years and maximum 15 years is more advantageous than group 2 in terms of benefits and harms in comparison to quarterly, biannual and annual screening strategies.

Group 1 biennial screening has a considerable reduction in number of CT scans, while, annual screening interval results in more detected patients with lower age at diagnosis than biennial screening interval but at the expense of double the number of CT scans. In group 2 screening start at age 50 years and end at age 74 years with smoking history of least 20 pack-years and quit year minimum 10 years and maximum 15 years. The comparative analysis between group 1 and group 2 showed that there is no significant difference on overall age at diagnosis and survival years of lung cancer patients but there are large differences in the number of patients screened and estimated number of CT scans as shown in Table 3. However, as the duration of screening intervals decreases, the number of CT scan will increase, which leads to additional cost and over diagnosis ratio, as well as increased radiation dose exposure. The screening is applied to high-risk individuals, not all of whom would develop lung cancer, and a more frequent screening will avert more deaths in those destined to develop LC but will cause more harm to those who are not. Frequent exposure to the radiation might cause other types of cancers. The analysis of simulated data shows that lung cancer screening strategies significantly reduced mortality and increased survival. Previous studies utilized similar strategies with different cohort effects to estimate mortality reduction [22, 48]. Observing the differences Group 1 offers a more efficient screening strategy in terms of time, cost, and radiation exposure.

Limitations: It seems that for Stage I at least, the more frequent screening finds cancer earlier. However, survival for screen-detected tumors is available for grouped sizes such as from 5 to 10 mm. For this reason, current study cannot model the differential survival benefit of finding a tumor of 6 mm versus 9 mm in size. The same limitation exists for all the size groups. This limits the resolution with which we can differentiate survivals. This leads to an apparently paradoxical effect that more frequent screening the patients may die (slightly) earlier, which is an artifact of the size groupings.

## 5. Conclusion

We used a previously developed and validated model of the natural progression and spontaneous detection of lung cancer, based on biologically and clinically sound assumptions: This model framework provided a platform to assess the outcome of secondary prevention strategies for clinical application, such as a periodic screening. Furthermore, we applied screening strategy with respect to smoking history to address the benefits and harms of frequent screening of high-risk individuals. This model suggests the optimal screening interval which could reduce cost and reduce radiation exposure without compromising survival of LC patients.

## Supporting information

S1 File This file (supplementary documents) includes S1 and S2 Tables and S1-S9 Figs, which provide additional data and analyses to support statements in manuscript. (DOCX)

## Abbreviation

CDF: Cumulative distribution function
CT: Computed tomography
DMS: Distant metastases size
LC: Lung Cancer
NCCN: National comprehensive cancer network
NMS: Nodal metastases size
NSCLC: Non-small cell lung cancer
NSCLC: non-small cell lung carcinoma
PTS: Primary tumor size
SCLC: Small cell lung carcinoma
SEER: Surveillance, Epidemiology and End Results
SHG: smoking history generator
TNM: Tumor node metastases
TSCE: Two-stage clonal expansion
TVDT: Tumor volume doubling time

## References

[1] Neal R. D. et al., “Stage, survival and delays in lung, colorectal, prostate and ovarian cancer: comparison between diagnostic routes,” *British Journal of General Practice*, vol. 57, no. 536, pp. 212–219, 2007. 17359608 PMC2042569

[2] Jemal A. et al., “Annual report to the nation on the status of cancer, 1975–2005, featuring trends in lung cancer, tobacco use, and tobacco control,” *JNCI*: *Journal of the National Cancer Institute*, vol. 100, no. 23, pp. 1672–1694, 2008. doi: 10.1093/jnci/djn389 19033571 PMC2639291

[3] Seyfried T. N. and Huysentruyt L. C., “On the origin of cancer metastasis,” *Critical Reviews™ in Oncogenesis*, vol. 18, no. 1–2, 2013. doi: 10.1615/critrevoncog.v18.i1-2.40 23237552 PMC3597235

[4] Sullivan L. B., Gui D. Y., and Vander Heiden M. G., “Altered metabolite levels in cancer: implications for tumour biology and cancer therapy,” *Nature Reviews Cancer*, vol. 16, no. 11, p. 680, 2016. doi: 10.1038/nrc.2016.85 27658530

[5] Losa F. et al., “2018 consensus statement by the Spanish Society of Pathology and the Spanish Society of Medical Oncology on the diagnosis and treatment of cancer of unknown primary,” *Clinical and Translational Oncology*, vol. 20, no. 11, pp. 1361–1372, 2018. doi: 10.1007/s12094-018-1899-z 29808414 PMC6182632

[6] DeVita V. T., Lawrence T. S., and Rosenberg S. A., *DeVita*, *Hellman*, *and Rosenberg’s cancer*: *principles & practice of oncology*. Lippincott Williams & Wilkins, 2008.

[7] Fick C. N. et al., “Genomic profiling and metastatic risk in early-stage non–small cell lung cancer,“ vol. 16, ed: Elsevier, 2023, pp. 9–16. 10.1016/j.xjon.2023.10.016 PMC10775106 38204702

[8] Li C. et al., “Advances in lung cancer screening and early detection,” *Cancer biology & medicine*, vol. 19, no. 5, p. 591, 2022. doi: 10.20892/j.issn.2095-3941.2021.0690 35535966 PMC9196057

[9] Rusch V. W. et al., “Occult metastases in lymph nodes predict survival in resectable non-small-cell lung cancer: report of the ACOSOG Z0040 trial,” (in eng), *J Clin Oncol*, vol. 29, no. 32, pp. 4313–9, Nov 10 2011, doi: 10.1200/JCO.2011.35.2500 21990404 PMC3221530

[10] Ohri N., Lu B., and Werner-Wasik M., “Occult nodal metastasis in non-small-cell lung cancer: implications for the radiation oncologist,” (in eng), *J Clin Oncol*, vol. 30, no. 19, p. 2423, Jul 1 2012, doi: 10.1200/JCO.2011.41.2148 22614992

[11] Rusch V. W. et al., “Occult metastases in lymph nodes predict survival in resectable non–small-cell lung cancer: report of the ACOSOG Z0040 trial,” *Journal of clinical oncology*, vol. 29, no. 32, pp. 4313–4319, 2011. doi: 10.1200/JCO.2011.35.2500 21990404 PMC3221530

[12] Chen X., Foy M., Kimmel M., and Gorlova O. Y., “Modeling the natural history and detection of lung cancer based on smoking behavior,” *PloS one*, vol. 9, no. 4, p. e93430, 2014. doi: 10.1371/journal.pone.0093430 24705368 PMC3976286

[13] Wood D. E. et al., “NCCN guidelines^®^ insights: lung cancer screening, version 1.2022: featured updates to the NCCN guidelines,” *Journal of the National Comprehensive Cancer Network*, vol. 20, no. 7, pp. 754–764, 2022. 35830884 10.6004/jnccn.2022.0036

[14] Foy M., Spitz M. R., Kimmel M., and Gorlova O. Y., “A smoking‐based carcinogenesis model for lung cancer risk prediction,” *International journal of cancer*, vol. 129, no. 8, pp. 1907–1913, 2011. doi: 10.1002/ijc.25834 21140453 PMC3116088

[15] Rosenberg M. A. et al., “Chapter 3: Cohort life tables by smoking status, removing lung cancer as a cause of death,” (in eng), *Risk Anal*, vol. 32 Suppl 1, pp. S25–38, Jul 2012, doi: 10.1111/j.1539-6924.2011.01662.x 22882890 PMC3594098

[16] Foy M., Yip R., Chen X., Kimmel M., Gorlova O. Y., and Henschke C. I., “Modeling the mortality reduction due to computed tomography screening for lung cancer,” *Cancer*, vol. 117, no. 12, pp. 2703–2708, 2011. doi: 10.1002/cncr.25847 21656748 PMC3138899

[17] Jeon J., Meza R., Krapcho M., Clarke L. D., Byrne J., and Levy D. T., “Chapter 5: Actual and counterfactual smoking prevalence rates in the US population via microsimulation,” *Risk Analysis*: *An International Journal*, vol. 32, pp. S51–S68, 2012. 10.1111/j.1539-6924.2011.01775.x PMC3478148 22882892

[18] Holford T. R. et al., “Patterns of birth cohort–specific smoking histories, 1965–2009,” *American journal of preventive medicine*, vol. 46, no. 2, pp. e31–e37, 2014. doi: 10.1016/j.amepre.2013.10.022 24439359 PMC3951759

[19] Kulama E., “Scanning protocols for multislice CT scanners,” (in eng), *Br J Radiol*, vol. 77 Spec No 1, pp. S2–9, 2004. [Online]. Available: http://www.ncbi.nlm.nih.gov/pubmed/15546838. doi: 10.1259/bjr/28755689 15546838

[20] Walter F., Ludig T., Iochum S., and Blum A., “Multi-detector CT in musculo-skeletal disorders,” (in eng), *JBR-BTR*, vol. 86, no. 1, pp. 6–11, Jan-Mar 2003. [Online]. Available: http://www.ncbi.nlm.nih.gov/pubmed/12675493. 12675493

[21] Wisnivesky J. P., Yankelevitz D., and Henschke C. I., “The effect of tumor size on curability of stage I non-small cell lung cancers,” *Chest*, vol. 126, no. 3, pp. 761–765, 2004. doi: 10.1378/chest.126.3.761 15364754

[22] Criss S. D., Sheehan D. F., Palazzo L., and Kong C. Y., “Population impact of lung cancer screening in the United States: projections from a microsimulation model,” *PLoS Medicine*, vol. 15, no. 2, p. e1002506, 2018. doi: 10.1371/journal.pmed.1002506 29415013 PMC5802442

[23] Duffy S. W. and Field J. K., “Understanding the lung cancer mortality reductions produced by low-dose CT screening—Authors’ reply,” *The Lancet Regional Health–Europe*, vol. 12, 2022. doi: 10.1016/j.lanepe.2021.100259 34950920 PMC8671110

[24] Black W. C., Keeler E. B., and Soneji S. S., “Cost-effectiveness of CT screening in the National Lung Screening Trial,” *The New England journal of medicine*, vol. 372, no. 4, pp. 388–388, 2015. doi: 10.1056/NEJMc1414726 25607437

[25] Schneider B. J., “Non-small cell lung cancer staging: proposed revisions to the TNM system,” *Cancer Imaging*, vol. 8, no. 1, p. 181, 2008. doi: 10.1102/1470-7330.2008.0029 18824424 PMC2556505

[26] Rami-Porta R. and Bolejack V., "Reply to “Inclusion of lymphangitis as a descriptor in the new TNM staging of lung cancer: Filling up the blank spaces”," *Journal of thoracic oncology*, vol. 10, no. 12, pp. e119–e120, 2015. 10.1097/JTO.0000000000000683 26709486

[27] Henschke C. I., Yip R., Yankelevitz D. F., and Miettinen O. S., “Computed tomography screening for lung cancer: prospects of surviving competing causes of death,” *Clinical lung cancer*, vol. 7, no. 5, pp. 323–325, 2006. doi: 10.3816/CLC.2006.n.013 16640803

[28] Ten Haaf K. et al., “A comparative modeling analysis of risk-based lung cancer screening strategies,” *JNCI*: *Journal of the National Cancer Institute*, vol. 112, no. 5, pp. 466–479, 2020. doi: 10.1093/jnci/djz164 31566216 PMC7225672

[29] Pastorino U. et al., “Prolonged lung cancer screening reduced 10-year mortality in the MILD trial: new confirmation of lung cancer screening efficacy,” *Annals of Oncology*, vol. 30, no. 7, pp. 1162–1169, 2019. doi: 10.1093/annonc/mdz117 30937431 PMC6637372

[30] Metwally E. M. et al., “Lung cancer screening in individuals with and without lung-related comorbidities,” *JAMA Network Open*, vol. 5, no. 9, pp. e2230146–e2230146, 2022. doi: 10.1001/jamanetworkopen.2022.30146 36066893 PMC9449784

[31] Flehinger B. J. and Kimmel M., “The natural history of lung cancer in a periodically screened population,” (in eng), *Biometrics*, Research Support, Non-U.S. Gov’t Research Support, U.S. Gov’t, P.H.S. vol. 43, no. 1, pp. 127–44, Mar 1987. [Online]. Available: http://www.ncbi.nlm.nih.gov/pubmed/3567302. 3567302

[32] Kimmel M. and Flehinger B. J., “Nonparametric estimation of the size-metastasis relationship in solid cancers,” (in eng), *Biometrics*, vol. 47, no. 3, pp. 987–1004, Sep 1991. [Online]. Available: http://www.ncbi.nlm.nih.gov/pubmed/1742451. 1742451

[33] Xu J. L. and Prorok P. C., “Estimating a distribution function of the tumor size at metastasis,” (in eng), *Biometrics*, vol. 54, no. 3, pp. 859–64, Sep 1998. [Online]. Available: http://www.ncbi.nlm.nih.gov/pubmed/9750239. 9750239

[34] Lin R. S. and Plevritis S. K., “Comparing the benefits of screening for breast cancer and lung cancer using a novel natural history model,” (in eng), *Cancer Causes Control*, vol. 23, no. 1, pp. 175–85, Jan 2012, doi: 10.1007/s10552-011-9866-9 22116537 PMC4146530

[35] Wood D. E. et al., “Lung cancer screening, version 3.2018, NCCN clinical practice guidelines in oncology,” *Journal of the National Comprehensive Cancer Network*, vol. 16, no. 4, pp. 412–441, 2018. doi: 10.6004/jnccn.2018.0020 29632061 PMC6476336

[36] Sverzellati N. et al., “Low-dose computed tomography for lung cancer screening: comparison of performance between annual and biennial screen,” *European radiology*, vol. 26, no. 11, pp. 3821–3829, 2016. doi: 10.1007/s00330-016-4228-3 26868497

[37] Field J. K. et al., “International association for the study of lung cancer computed tomography screening workshop 2011 report,” *Journal of Thoracic Oncology*, vol. 7, no. 1, pp. 10–19, 2012. doi: 10.1097/JTO.0b013e31823c58ab 22173661

[38] Bach P. B. et al., “Benefits and harms of CT screening for lung cancer: a systematic review,” *Jama*, vol. 307, no. 22, pp. 2418–2429, 2012. doi: 10.1001/jama.2012.5521 22610500 PMC3709596

[39] McKee B. J. et al., “Experience with a CT screening program for individuals at high risk for developing lung cancer,” *Journal of the American College of Radiology*, vol. 13, no. 2, pp. R8–R13, 2016. 26846536 10.1016/j.jacr.2015.12.006

[40] Lillie S. E. et al., “What factors do patients consider most important in making lung cancer screening decisions? Findings from a demonstration project conducted in the Veterans Health Administration,” *Lung Cancer*, vol. 104, pp. 38–44, 2017. doi: 10.1016/j.lungcan.2016.11.021 28212998

[41] de Koning H. J. et al., “Benefits and harms of computed tomography lung cancer screening strategies: a comparative modeling study for the US Preventive Services Task Force,” *Annals of internal medicine*, vol. 160, no. 5, pp. 311–320, 2014. 24379002 10.7326/M13-2316 PMC4116741

[42] Becker N. et al., “Lung cancer mortality reduction by LDCT screening—Results from the randomized German LUSI trial,” *International journal of cancer*, vol. 146, no. 6, pp. 1503–1513, 2020. doi: 10.1002/ijc.32486 31162856

[43] Team N. L. S. T. R., “Reduced lung-cancer mortality with low-dose computed tomographic screening,” *New England Journal of Medicine*, vol. 365, no. 5, pp. 395–409, 2011. doi: 10.1056/NEJMoa1102873 21714641 PMC4356534

[44] Huo J., Shen C., Volk R. J., and Shih Y.-C. T., “Use of CT and chest radiography for lung cancer screening before and after publication of screening guidelines: intended and unintended uptake,” *JAMA internal medicine*, vol. 177, no. 3, pp. 439–441, 2017. doi: 10.1001/jamainternmed.2016.9016 28135349 PMC5893328

[45] Moyer V. A., “Screening for lung cancer: US Preventive Services Task Force recommendation statement,” *Annals of internal medicine*, vol. 160, no. 5, pp. 330–338, 2014. 24378917 10.7326/M13-2771

[46] Wender R. et al., “American Cancer Society lung cancer screening guidelines,” *CA*: *a cancer journal for clinicians*, vol. 63, no. 2, pp. 106–117, 2013. doi: 10.3322/caac.21172 23315954 PMC3632634

[47] Meza R. et al., “Evaluation of the benefits and harms of lung cancer screening with low-dose computed tomography: modeling study for the US Preventive Services Task Force,” *Jama*, vol. 325, no. 10, pp. 988–997, 2021. doi: 10.1001/jama.2021.1077 33687469 PMC9208912

[48] McMahon P. M. et al., “Comparing benefits from many possible computed tomography lung cancer screening programs: extrapolating from the National Lung Screening Trial using comparative modeling,” *PloS one*, vol. 9, no. 6, p. e99978, 2014. doi: 10.1371/journal.pone.0099978 24979231 PMC4076275

