# Supplementary material for: Occult metastases and survival of lung cancer by clinical diagnosis and CT screening: A simulation study
Source: PLoS One. 2025 Jan 3;20(1):e0313544. doi: 10.1371/journal.pone.0313544 (PMC11698447; doi:10.1371/journal.pone.0313544)
Supplement: S1 File — (DOCX) [file pone.0313544.s001.docx]

**Supplementary Document**

**Occult Metastases and Survival of Lung Cancer by Clinical Diagnosis and CT Screening: A Simulation Study**

**Running title**  Occult Metastasis and Survival of LC

Xing Chen^1,2^, Ghulam Muhammad Kanhar^2^, Songli Hu^3^, Chaomin Wu^4^, Guanqun Chao^1*^, Mengqi Jing^1^, Fengjiang Zhang^3*^, Millennia Young^6^, Marek Kimmel^7^, Liying Chen^1*^, Olga Y. Gorlova^5^

^1^Zhejiang Sir Run Run Shaw Hospital, Department of Medicine, Zhejiang University, Hangzhou, China. ^2^Department of Biomedical Engineering, Key Laboratory of Biomedical Engineering of Ministry of Education of China, Zhejiang University, 38 Zheda Road, Zhou Yi Qing Building, Hangzhou, Zhejiang, 310027, China; ^3^Department of Anesthesiology, Second Affiliated Hospital, Zhejiang University, Hangzhou, Zhejiang, China; ^4^Department of Anesthesiology, The Fourth Affiliated Hospital, International lnstitutes of Medicine，Zhejiang University School of Medicine, Yiwu, China. ^5^Department of Medicine Epidemiology and Population Sciences, Baylor College of Medicine, Houston Texas, USA; ^6^Human Health and Performance Directorate / Biomedical Research & Environmental Sciences Division, NASA Johnson Space Center, 2101 NASA Parkway, Houston, TX77058 United States; ^7^Departments of Statistics and Bioengineering, Rice University, 6100 Main Street, Houston, Texas, 77251, USA;

* Corresponding author: Fengjiang Zhang, Guanqun Chao and Liying Chen

Address: Zhejiang Sir Run Run Shaw hospital affiliated to Zhejiang medical school, Qingchun east road #3, Hangzhou, Zhejiang, China.

Tel/Fax: +86057186002113;

Cell: +86 13588706741.

Email: 3197020@zju.edu.cn, chaoguanqun@zju.edu.cn

Authors Email: cnxingchen@zju.edu.cn (X.C), munaim.gm@gmail.com (GM.K), 22118652@zju.edu.cn (S.H), cmwu@zju.edu.cn (C.W), chaoguanqun@zju.edu.cn (G.C), 3319065@zju.edu.cn (M.J), zrzfj@zju.edu.cn (F.Z), millennia.young@nasa.gov (M.Y), kimmel@rice.edu (M.K), 3197020@zju.edu.cn (L.C) olga.gorlova@bcm.edu (G.O.Y)

**Supplementary materials**

**Supplementary. Table 1.** Parameters of the response functions used in the model framework (Page 2)

**Supplementary. Table 2.** the classification of detected stage (Page3)

**Survival Plot S1.** Stage 1 tumor size base survival function (Page4)

**Survival Plot S2.** Stage 2 tumor size base survival function (Page4)

**Survival Plot S3.** Stage 3 tumor size base survival function (Page5)

**Survival Plot S4.** Stage 4 tumor size base survival function (Page5)

**Survival Plot S5.** 5- Year survival plot between simulated data, baseline and SEER (page6)

**Survival Plot S6.** 15-year survival plot between simulated data, baseline and SEER (Page 6)

**Mortality Plot S7.** Group 1 and Grou2 Mortality reduction (Page 7)

**Mortality Plot S8.** Group 1 Mortality reduction plot (Page 8)

**Mortality Plot S9.** Group 2 Mortality reduction plot (Page 8)

**Supplementary Table 1.** Parameters of the response functions used in the model framework

| Parameter* | ξ | µ_n_ | µ_m_ | K | θ | η | W_0_ | W_1_ | W_2_ |
| --- | --- | --- | --- | --- | --- | --- | --- | --- | --- |
| Estimate | 0.01 | 8.05×10^-9^ | 2.78×10^-9^ | 3.8 | 1.15 | 1.0×10^-4^ | 0.065 | 1.50×10^3^ | 7.00×10^4^ |

*Parameters were estimated using the data from Surveillance, Epidemiology and End Results (SEER)

**Supplementary Table 2.**  The classification of detected stage

| T (Primary Tumor, cm) | N (Regional Lymph Nodes, cm) | M (Distant Metastasis, cm) |
| --- | --- | --- |
| T0: 0 ≤ PTS ≤ 0.0618; | N0: NMS = 0; | M0: DMS= 0; |
| T1a: 0.0618 < PTS ≤ 2; | N1: 0< NMS ≤ 0.4; | M1: DMS > 0; |
| T1b: 2 < PTS ≤ 3; | N2: 0.4< NMS ≤ 5; | |
| T2a: 3 < PTS ≤ 5; | N3: NMS > 5; |  |
| T2b: 5 < PTS ≤ 7; |  |  |
| T3: 7 < PTS ≤ 12; |  |  |
| T4: PTS > 12; |  | |

* The stage classification was based on the Anatomic stage/prognostic groups;

** This is detection limit of CT screen. We assumed that if the tumor size was lower than 0.0618, the detection limit, the person would be considered as having no metastases. Considering occult metastasis: N0: 0 ≤ NMS ≤ 0.0618; N1:0.0618< NMS ≤ 0.4; N2: 0.4< NMS ≤ 5; N3: NMS > 5; and M0: 0 ≤ DMS ≤ 0.0618; M1: DMS > 0.0618.

**Survival Plot**

We calculated the survival function based on extent of disease (EOD) tumor size. It records the largest dimension of the primary tumor in millimeters. The survival function plots are given below.

**
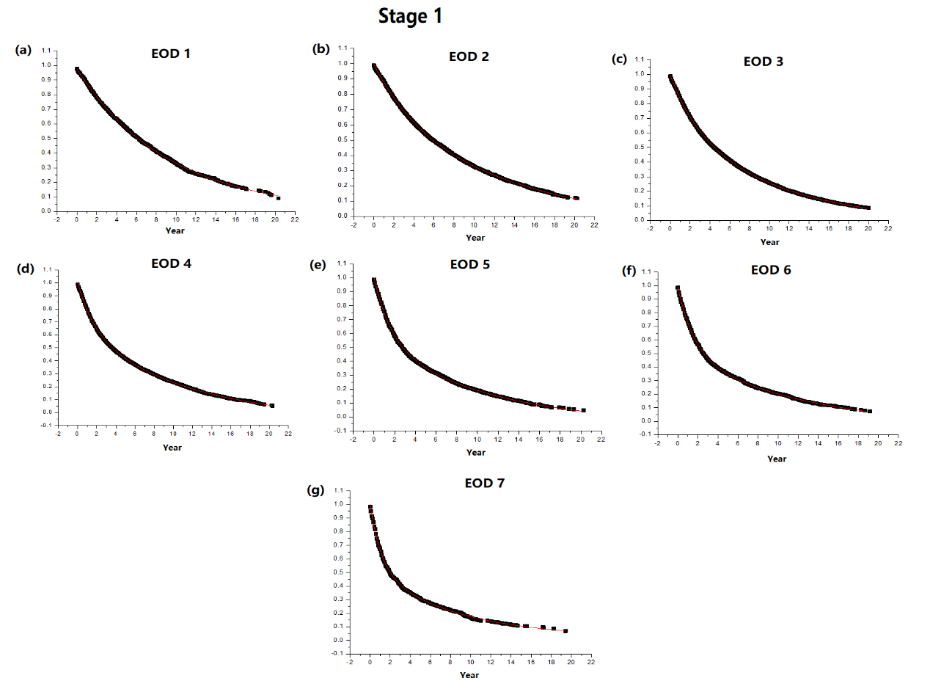
**

**S1 Figure 1.** The survival function stratified based on tumor size stage 1.

**
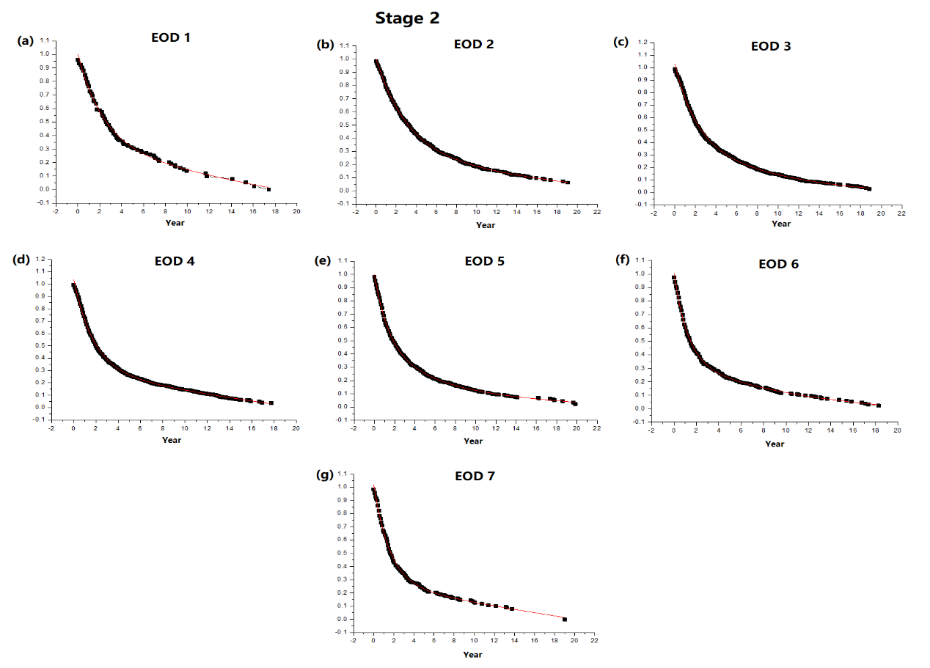
**

**S2 Figure 2.** The survival function stratified based on tumor size stage 2.

**
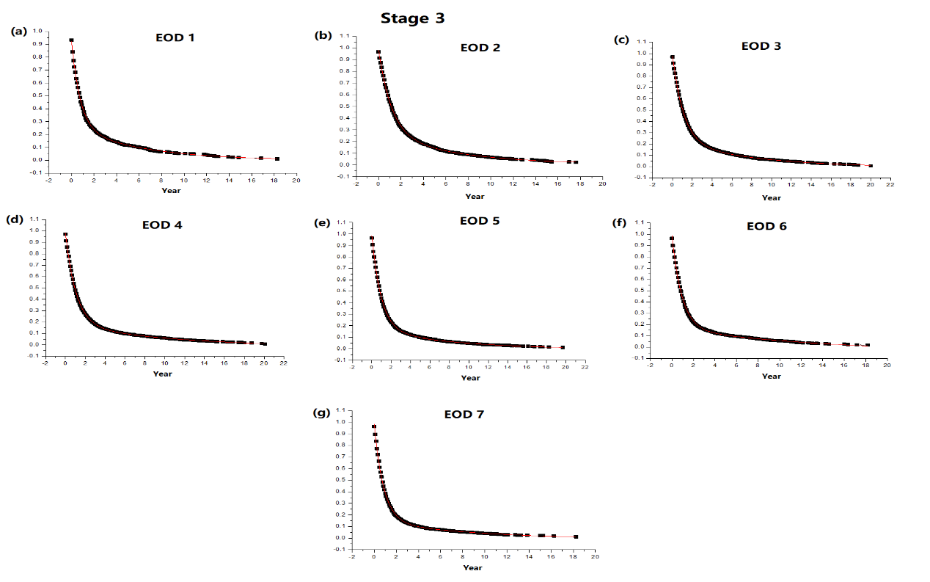
**

**S3 Figure 3.** The survival function stratified based on tumor size stage 3.**
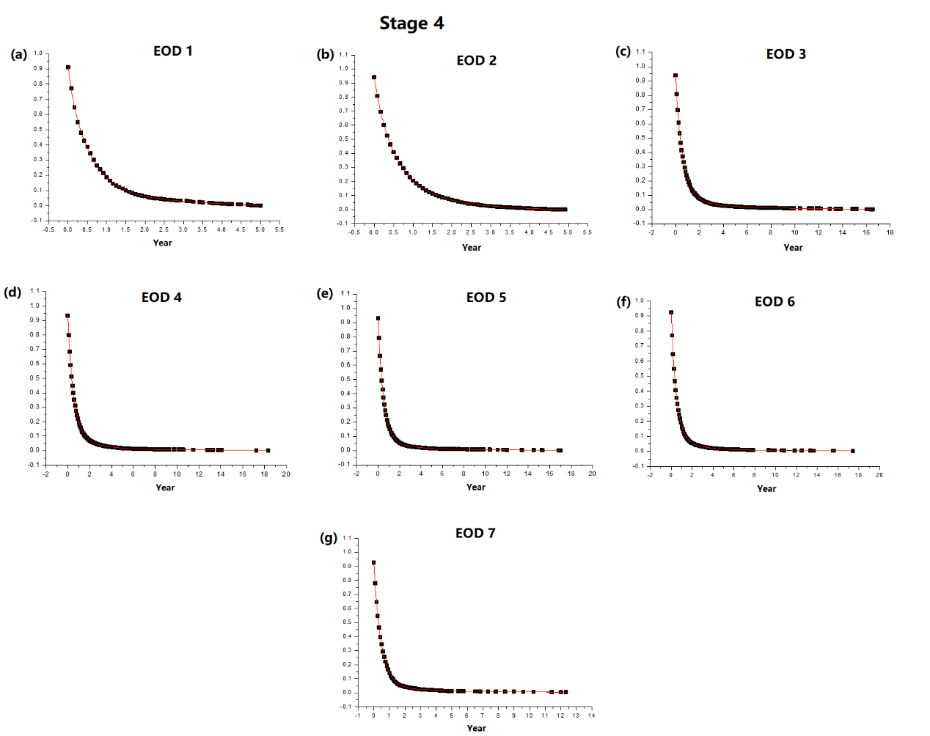
**

**S4 Figure 4.** The survival function stratified based on tumor size stage 4

To witness the difference between SEER and stage based data we did a comparative analysis between simulated data, baseline and SEER.


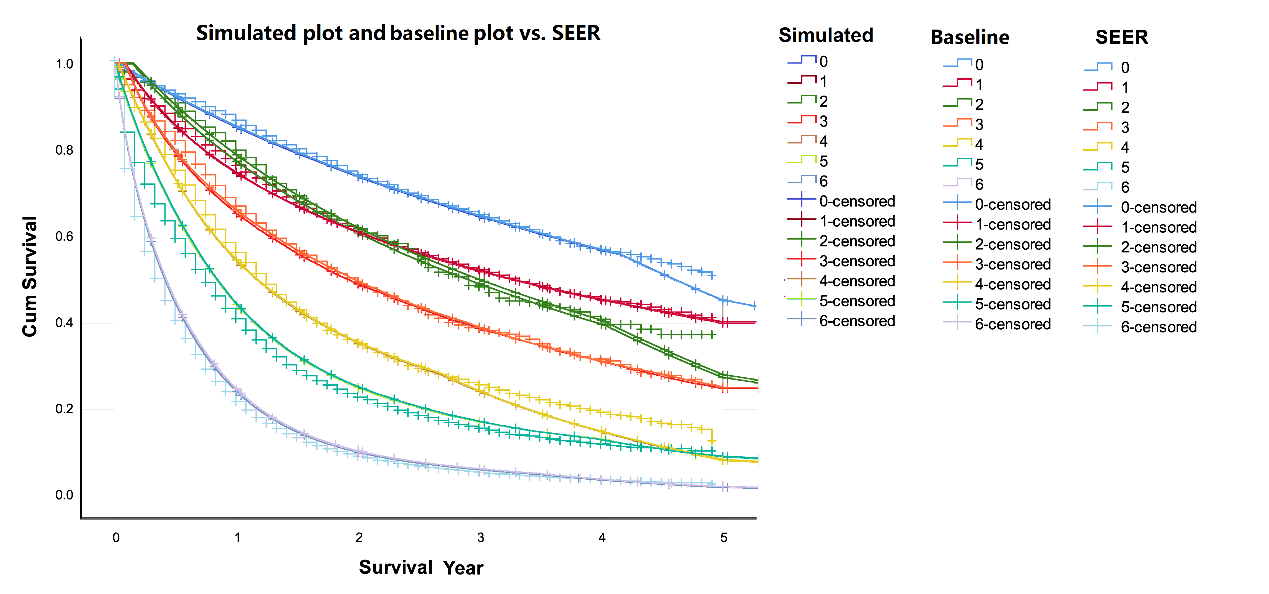


S5 Figure. 5. Comparative analysis of stage wise 5-year survival between simulated data, baseline and SEER.

Furthermore, to witness the fifteen-year overall survival difference between SEER, baseline and tumor size data.


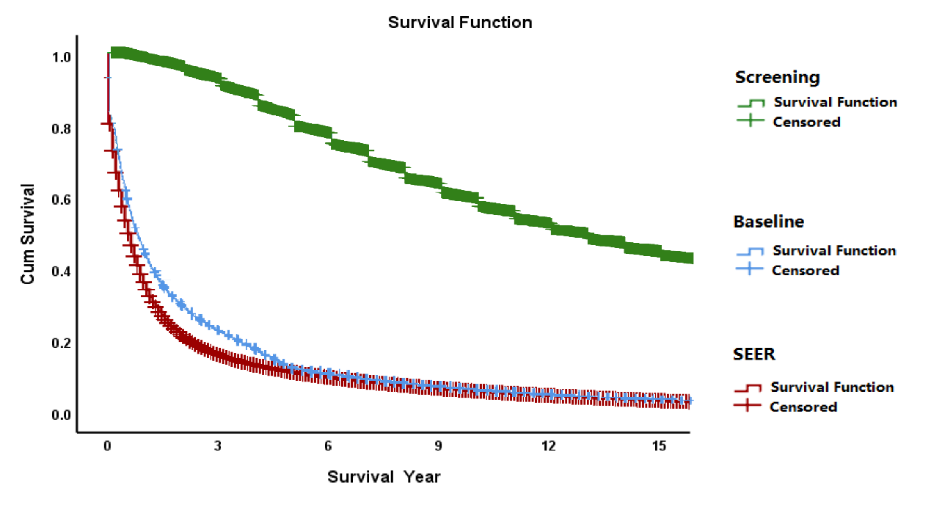


S6 Figure 6. 15-year survival difference between SEER, baseline and screening.

**Mortality Plot:** The simulated data screening intervals projected annual mortality reduction for group 1 which peaked in 1980 at 33.58% and decreased to 2.06% by 1999 and group 2 in 1980 at 41.07% and decreased to 3.69% by 1999. Whereas, overall each screening intervals of group 1 and group 2 estimated mortality reduction between 15.04% to 19.82%.


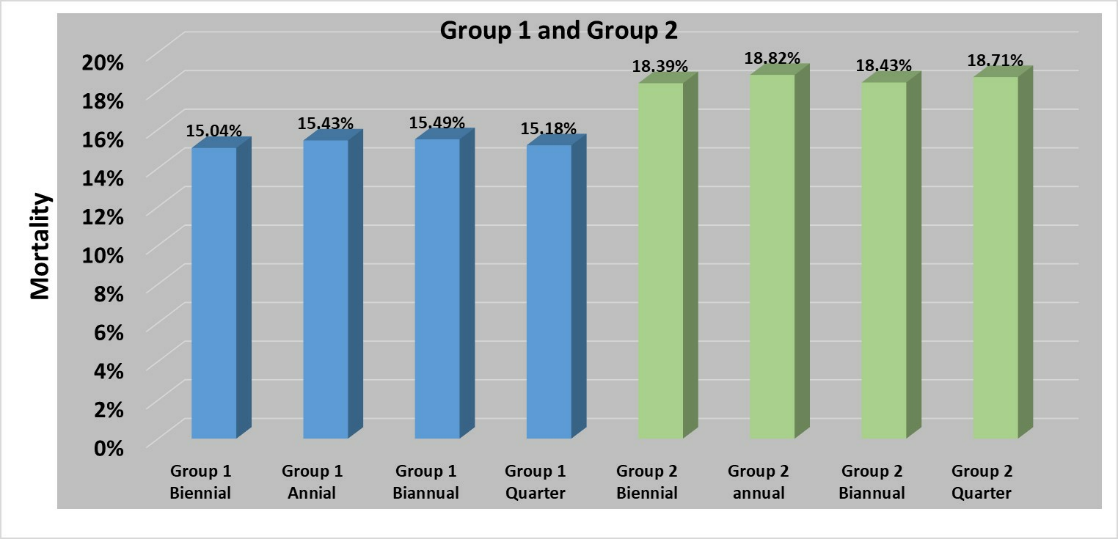


S7 Figure 7. The mortality reduction estimates of group 1 and group 2 quarter, biannual, annual and biennial screening intervals.


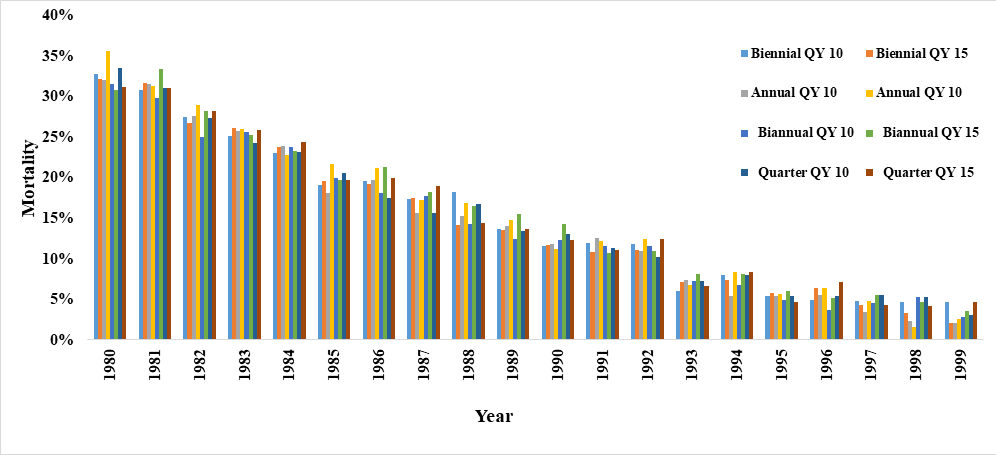


S8 Figure 8. Group 1. Projected mortality reduction for the lung cancer screening study population on an annual basis (1980 to 1999). QY*denotes the smoking quit duration (years).


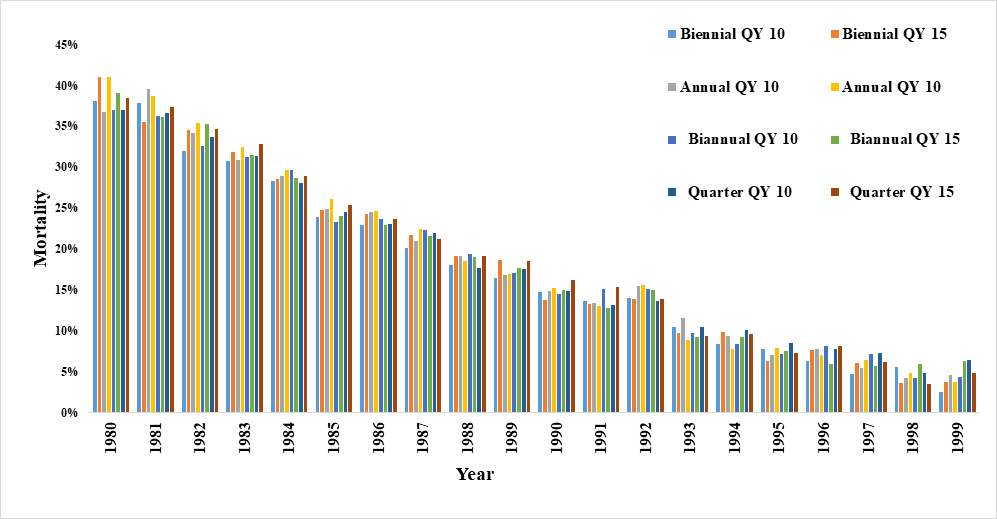


S9 Figure 9. Group 2. Projected mortality reduction for the lung cancer screening study population on an annual basis (1980 to 1999). QY*denotes the smoking quit duration (years).

The reason behind this mortality reduction is that we set screening strategy starting in year 1980 for those age 55 years and above. The people who were born before and after 1925 have substantially different diagnosed stages and survival years. Most of the people born before 1925 were diagnosed with large tumors and have shorter lifespans than people born after 1925. The screened people lived longer as the figure S3 and S4 show the drop of mortality in accordance to screening strategies.
